# Характеристика активной субстанции препарата дрожжей Saccharomyces сerevisiae, обладающей радиопротекторными свойствами

**DOI:** 10.18699/VJ20.658

**Published:** 2020-10

**Authors:** G.S. Ritter, V.P. Nikolin, N.A. Popova, A.S. Proskurina, P.E. Kisaretova, O.S. Taranov, T.D. Dubatolova, E.V. E.V. Dolgova, E.A. Potter, S.S. Kirikovich, Y.R. Efremov, S.I. Bayborodin, M.V. Romanenko, M.I. Meschaninova, A.G. Venyaminova, N.A. Kolchanov, S.S. Bogachev

**Affiliations:** Institute of Cytology and Genetics of Siberian Branch of the Russian Academy of Sciences, Novosibirsk, Russia; Institute of Cytology and Genetics of Siberian Branch of the Russian Academy of Sciences, Novosibirsk, Russia; Institute of Cytology and Genetics of Siberian Branch of the Russian Academy of Sciences, Novosibirsk, Russia Novosibirsk State University, Novosibirsk, Russia; Institute of Cytology and Genetics of Siberian Branch of the Russian Academy of Sciences, Novosibirsk, Russia; Institute of Cytology and Genetics of Siberian Branch of the Russian Academy of Sciences, Novosibirsk, Russia; State Research Center of Virology and Biotechnology “Vector”, Koltsovo, Novosibirsk region, Russia; Institute of Molecular and Cellular Biology of Siberian Branch of the Russian Academy of Sciences, Novosibirsk, Russia; Institute of Cytology and Genetics of Siberian Branch of the Russian Academy of Sciences, Novosibirsk, Russia; Institute of Cytology and Genetics of Siberian Branch of the Russian Academy of Sciences, Novosibirsk, Russia; Institute of Cytology and Genetics of Siberian Branch of the Russian Academy of Sciences, Novosibirsk, Russia; Institute of Cytology and Genetics of Siberian Branch of the Russian Academy of Sciences, Novosibirsk, Russia Novosibirsk State University, Novosibirsk, Russia; Institute of Cytology and Genetics of Siberian Branch of the Russian Academy of Sciences, Novosibirsk, Russia Novosibirsk State University, Novosibirsk, Russia; Novosibirsk State University, Novosibirsk, Russia; Institute of Chemical Biology and Fundamental Medicine of Siberian Branch of the Russian Academy of Sciences, Novosibirsk, Russia; Institute of Chemical Biology and Fundamental Medicine of Siberian Branch of the Russian Academy of Sciences, Novosibirsk, Russia; Institute of Cytology and Genetics of Siberian Branch of the Russian Academy of Sciences, Novosibirsk, Russia; Institute of Cytology and Genetics of Siberian Branch of the Russian Academy of Sciences, Novosibirsk, Russia

**Keywords:** double-stranded RNA, B-190, spleen colonies, double-stranded breaks, двуцепочечная РНК, Б-190, селезеночные колонии, двуцепочечные разрывы

## Abstract

В работе охарактеризованы некоторые биологические особенности радиопротекторного действия препарата двуцепочечной РНК. Обнаружено, что препарат дрожжевой РНК обладает пролонгированным радиопротекторным действием при облучении животных летальной дозой в 9.4 Гр. При облучении через 1 ч и на 4-е сутки после введения 7 мг препарата РНК выживает 100 % животных на 70-е сутки наблюдения, при облучении на 8-е и 12-е сутки – 60 % животных. Были оценены временные параметры процесса репарации двуцепочечных разрывов, индуцированных γ-лучами. Выявлено, что введение препарата РНК в момент максимального количества двуцепо- чечных разрывов, через 1 ч после облучения, снижает эффективность радиопротекторного действия по сравнению с введением за 1 ч до облучения и через 4 ч после облучения. Проведено сравнение эффективности радиозащит- ного действия штатного радиопротектора Б-190 и препарата РНК в одном эксперименте. Установлено, что препарат суммарной РНК не уступает по эффективности препарату Б-190. Выживаемость на 40-е сутки после облучения для группы мышей, получавших препарат РНК, составила 78 %, для Б-190 – 67 % животных. В ходе аналитических иссле- дований препарата суммарной РНК дрожжей обнаружилось, что препарат представляет собой смесь одноцепочеч- ной и двуцепочечной РНК. Радиопротекторными свойствами обладает только двуцепочечная РНК. При введении 160 мкг препарата двуцепочечной РНК выживает 100 % подопытных животных после абсолютно летальной дозы гамма-радиации 9.4 Гр. Установлено, что радиозащитный эффект двуцепочечной РНК зависит не от последователь- ности, а от ее двуцепочечной формы, причем для осуществления радиопротекторного действия двуцепочечная РНК должна иметь «открытые» концы молекулы. Предполагается, что радиозащитное действие препарата двуцепочеч- ной РНК связано с участием молекул РНК в корректном восстановлении поврежденного облучением хроматина в стволовых клетках крови. Сохранившие жизнеспособность стволовые гемопоэтические клетки мигрируют на пери- ферию и достигают селезенки, где активно пролиферируют. Вновь образовавшаяся клеточная популяция восстанав- ливает кроветворную и иммунную системы, что определяет выживание летально облученных животных.

## Abstract (en)

The paper describes some biological features of the radioprotective effect of double-stranded RNA preparation. It was found that yeast RNA preparation has a prolonged radioprotective effect after irradiation by a lethal dose of 9.4 Gy. 100 % of animals survive on the 70th day of observation when irradiated 1 hour or 4 days after 7 mg RNA preparation injection, 60 % animals survive when irradiated on day 8 or 12. Time parameters of repair of double-stranded breaks induced by gamma rays were estimated. It was found that the injection of the RNA preparation at the time of maximum number of double-stranded breaks, 1 hour after irradiation, reduces the efficacy of radioprotective action compared with the injection 1 hour before irradiation and 4 hours after irradiation. A comparison of the radioprotective effect of the standard radioprotector B-190 and the RNA preparation was made in one experiment. It has been established that the total RNA preparation is more efficacious than B-190. Survival on the 40th day after irradiation was 78 % for the group of mice treated with the RNA preparation and 67 % for those treated with B-190. In the course of analytical studies of the total yeast RNA preparation, it was found that the preparation is a mixture of single-stranded and double-stranded RNA. It was shown that only double-stranded RNA has radioprotective properties. Injection of 160 μg double-stranded RNA protects 100 % of the experimental animals from an absolutely lethal dose of gamma radiation, 9.4 Gy. It was established that the radioprotective effect of double-stranded RNA does not depend on sequence, but depends on its double-stranded form and the presence of “open” ends of the molecule. It is supposed that the radioprotective effect of double-stranded RNA is associated with the participation of RNA molecules in the correct repair of radiation-damaged chromatin in blood stem cells. The hematopoietic pluripotent cells that have survived migrate to the periphery, reach the spleen and actively proliferate. The newly formed cell population restores the hematopoietic and immune systems, which determines the survival of lethally irradiated animals.

## Введение

Ионизирующее излучение воздействует на живой орга- низм таким образом, что приводит к повреждению его функциональных систем и гибели. В настоящее время считается, что ионизирующее излучение оказывает наи- большее воздействие на мембранные структуры и ядро клетки. Лизис мембран приводит к разрушению структуры клетки, а дефекты в ядерной ДНК ведут к нарушению интегральной функциональной целостности хроматина, атипическому течению клеточного деления, появлению хромосомных аберраций и апоптозу (Dent et al., 2003). Основными клетками-мишенями для гамма-лучей явля- ются низкодифференцированные клетки костного мозга, зародышевые клетки семенников, кишечный и кожный эпителий (Bergonié, Tribondeau, 2003; Vogin, Foray, 2013). Радиочувствительность всего организма у млекопитающих приравнивается к радиочувствительности кроветворных клеток, так как их аплазия, возникающая после общего облучения минимальной абсолютно смертельной дозой, приводит к гибели организма.

Под радиозащитным или радиопротекторным эффек- том понимают снижение частоты и тяжести постлучевых повреждений биомолекул и(или) стимуляцию процессов их пострадиационной репарации. Наиболее эффективные радиопротекторы относятся к двум классам химических соединений (Patt et al., 1949; Fridovich, 1995). Это серосодержащие радиозащитные вещества (аминотиолы), выполняющие функцию «молекулярных ловушек» свобод- ных радикалов, и производные индолилалкиламинов: аго- нисты биологически активных аминов, способные через специфические клеточные рецепторы вызывать острую гипоксию и угнетение метаболизма в радиочувствитель- ных тканях (Ward, 1988; Dent et al., 2003; Wang et al., 2013).

Как было сказано ранее, наиболее губительное воздей- ствие ионизирующее излучение оказывает на молекулу ДНК ядерного хроматина. Повреждения хромосом, сле- дующий за этим аберрантный митоз и гибель клетки – это еще один механизм циторедуцирующего действия ионизирующего излучения. При воздействии активных мета- болитов на ДНК хроматина возникают все возможные из описанных в литературе повреждений этих молекул. Наиболее фатальными считаются двуцепочечные разры- вы (ДЦР). Если в клетке нарушены системы репарации таких повреждений, то клетка запускает механизмы само- уничтожения.

В настоящем исследовании описывается новый прин- цип радиопротекторного действия, не связанный с про- текцией от непосредственно γ-кванта и с ограничением воздействия оксидативного стресса, вызываемого вто- ричными радикалами, а характеризующийся успешным пострадиационным восстановлением стволовых гемопоэтических предшественников, обусловленным участи- ем в репаративном процессе фрагментов экстраклеточной двуцепочечной нуклеиновой кислоты. Такое введение в репаративный процесс внешнего «корректора» в конечном итоге определяет восстановление кроветворной и иммунной системы и сохранение жизнеспособности об- лученного организма.

## Материалы и методы

**Животные.** В работе были использованы трехмесячные мыши линий CBA/Lac, C57BL и CC57BR (самцы и сам- ки, 18–22 г) разведения вивария Института цитологии и генетики (ИЦиГ) СО РАН. Животные содержались в группах по 6–10 мышей на клетку со свободным доступом к пище и воде.

**Облучение экспериментальных животных** проводили на γ-установке (источник Cs137 ИГО 1, Россия) до- зой 9.4 Гр при мощности дозы 0.74–1.4 Гр/мин. Подопытных и контрольных мышей облучали группами по 9–10 животных в контейнере размером 20 × 20 × 40 см. Радиопротекторное действие препарата суммарной РНК дрожжей (НПО «Биолар», Россия) оценивали по гибели экспериментальных животных в промежуток времени до 30–90 сут. Препарат суммарной РНК дрожжей и двуцепо- чечная РНК вводились мышам однократно внутривенно до облучения в количестве, отдельно указанном для каждого эксперимента. Оценка количества селезеночных колоний после фиксации органа в 4 % параформальдегиде проводилась на 9–12-е сутки после облучения.

**Выделение фракций препарата РНК.** Хроматогра- фию препарата РНК выполняли на колонке объемом 10 мл, диаметром 1 см. Сухой гидроксиапатит (ГАП) подвергали набуханию в 10 мл воды, после чего заполняли колонку. Колонку промывали 30 мл 0.01 М PBS. Наносили на колонку раствор РНК и промывали 30 мл 0.01 М PBS. Элюировали РНК 0.15 М PBS, затем промывали 0.18 М PBS и повторно элюировали нуклеиновые кислоты 0.25 М PBS. Полученный при хроматографии раствор РНК в PBS диализовали против ТЕ-буфера (H2O, обработанная DEPC; 10 мM Tris-HCl, 10 мM ЭДТА, pH 7.4) при +4 °С в течение суток с двумя сменами буфера. Электрофорез препаратов нуклеиновых кислот проводился в 1 % или 1.5 % агарозном геле с содержанием 2 мкг/мл бромистого этидия в трис-ацетатном буфере.

**Выделение клеток костного мозга.** Клетки костного мозга мышей вымывали из трубчатых костей средой RPMI-1640, тщательно ресуспендировали. Суспензию аккуратно наслаивали на 3 мл смеси фикол-урографин (15 % урографин, 7 % фиколл, ρ = 1.119), центрифуги- ровали при 400 g, 4 °С в течение 40 мин. После центри- фугирования вся клеточная масса разделялась на клетки, составляющие интерфазное кольцо (мононуклеары) и осадок. Мононуклеары отбирали в новую пробирку, про- мывали 4 мл RPMI-1640 и осаждали центрифугированием при 400 g, 4 °С в течение 5 мин.

**Анализ репаративного цикла в клетках костного мозга.** Через 30, 60 и 120 мин после облучения мышей линии СВА абсолютно летальной дозой 9.4 Гр из труб- чатых костей выделяли клетки костного мозга. Анализ репаративного цикла по количеству ДЦР осуществляли методом «кометных хвостов» или при помощи антител к гистону γ-H2aX, как описано в работе (Dolgova et al., 2014). Длину «кометных хвостов» (TM – tail moment) оценивали в программе CASP и ImageJ. Образцы, окрашенные антителами к гистону γ-H2aX, анализировали при помо- щи проточного цитофлуориметра BD FACSAria в ЦКП проточной цитофлуорометрии ИЦиГ СО РАН. Значения, полученные после анализа 50–100 кометных хвостов, были нормированы к показателям, определенным в 30-й минуте, и усреднены.

**Патоморфологический анализ органов.** Органы фиксировались в 4 % формальдегиде и заливались в парафиновые блоки. Парафиновые срезы проводились через серию спиртов и окрашивались гематоксилином-эозином.

**Сравнение эффективности радиопротекторного дей- ствия препарата суммарной РНК дрожжей и штатного радиопротектора Б-190.** В качестве препарата сравнения использовали радиопротектор Б-190 (ФГУП НПЦ «Фарм- защита» ФМБА России). Препарат Б-190 вводили мышам за 20 мин до облучения перорально в количестве 2.5 мг/ мышь в объеме 0.25 мл. Препарат суммарной РНК вводили мышам за 60 мин до облучения внутривенно в количестве 7 мг/мышь в объеме 0.5 мл (0.15 М PBS). Сравнивали выживаемость животных после облучения дозой 9.4 Гр, делали патоморфологический анализ органов.

**Качественная реакция на ДНК (реакция Дише).** Метод основан на способности дезоксирибозы образовывать соединение синего цвета с дифениламином при нагрева- нии в среде, содержащей смесь ледяной уксусной и кон- центрированной серной кислот (Dische, 1957). С рибозой РНК аналогичная реакция дает зеленое окрашивание. Дифениламиновый реактив представляет собой 1 % (W/V) раствор дифениламина в смеси ледяной уксусной кисло- ты и 2.75 % (W/V) концентрированной серной кислоты ( р20 = 1.836). К осадку нуклеиновых кислот добавляют 0.5 мл раствора едкого натра (0.1 М) и приливают равный объем дифениламинового реактива. Раствор нагревают в течение 15–20 мин на кипящей водяной бане. Появляется характерное для субстрата окрашивание.

**Клонирование кДНК копий молекул РНК, элюирующихся с ГАП 0.25 М PBS.** Для получения кДНК с РНК использовали систему ревертазного синтеза и набор DOP- PCR master kit («Медиген»). кДНК копии клонировали в плазмидном векторе Bluescript (НПО «Вектор») после «полировки» концов фрагментов с помощью Pfu полимеразы и трансформировали в электрокомпетентные клетки XL1-Blue MRF. Штамм E. coli XL1-Blue MRF любезно предоставлен лабораторией иммуногенетики Института молекулярной и клеточной биологии СО РАН. После анализа электрофоретической подвижности ДНК, вы- деленной из полученных трансформантов, отобранные клоны были секвенированы с использованием протокола фирмы Applied Biosystems (США) при помощи автомати- ческого ДНК секвенатора Applied Biosystems 3500 Genetic Analyzer с 8-канальным капиллярным блоком. Секвениро- ванные клоны анализировались в программе Vector NTI. Последовательности были выравнены и собраны в группы гомологии. Контекстный анализ проводили на сайте http://genome.ucsc.edu, используя инструмент Blat.

## Результаты

**Радиопротекторное действие суммарной дрожжевой РНК**

Было проанализировано 10 различных серийных препаратов дрожжевой суммарной РНК на ее способность защищать животных от летальной дозы γ-радиации. Об- наружено, что радиозащитный эффект препарата прямо не связан с процентным содержанием РНК и белка в препарате. Оценена длительность радиозащитного дей- ствия препарата. Для этого очищенная стерильная РНК дрожжей в количестве 7 мг вводилась экспериментальным мышам линии C57BL за час, за сутки, за 4, 8 и 12 сут до облучения летальной дозой радиации 9.4 Гр. Оказалось, при облучении через 1 ч и на 4-е сутки от введения пре- парата РНК выживает 100 % животных на 70-е сутки наблюдения, при облучении на 8-е и 12-е сутки – 60 % животных (Риттер и др., 2018).

Состояние экспериментальных животных, выживших после летальных доз γ-радиации в отдаленные сроки по- сле проведенного облучения, свидетельствовало о значи- тельных нарушениях в клетках, формирующих кожный покров мышей. В течение 50–150 дней после обработки мыши прогрессивно седели (рис. 1, а). Анализ развития селезеночных колоний после облучения, проведенного на 9–12-е сутки после воздействия, предполагал, что главной мишенью воздействия препарата РНК являлись стволовые клетки крови костного мозга (см. рис. 1, б ). Спасенные от разрушения ионизирующим облучением гемопоэтические предшественники способны выходить на периферию и заполнять опустошенные вследствие обработки радиа- цией иммунокомпетентные органы, например селезенку. В результате мобилизации спасенных стволовых клеток крови в белой пульпе селезенки формируются селезеноч- ные колонии, из которых развивается новая иммунная и кроветворная системы организма взамен разрушенных радиацией.

**Fig. 1.**
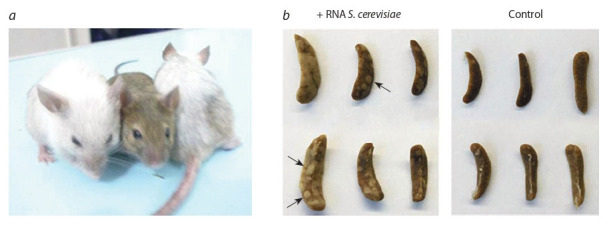
Characterization of the radioprotective effect of RNA after irradiation of mice with the absolute lethal radiation dose 9.4 Gy. a – phenotypic comparison of CBA mice irradiated one hour after injection of RNA preparation (left and right) and an intact mouse (center) 150 days after irradiation; b – Spleen colonies detected on days 9–12 after irradiation in animals treated with the RNA preparation and in control animals. Arrows indicate leukocyte colonies, which are the criterion for the survival of experimental mice.

**Радиотерапевтическое действие препарата суммарной РНК дрожжей, ориентированное на временные параметры цикла репарации ДЦР в клетках костного мозга мышей, индуцированных гамма радиацией**

Известно, что γ-радиация индуцирует разрушение хрома- тина в клетках костного мозга и в том числе в стволовых клетках крови, что и приводит к развитию лучевой болез- ни и гибели организма (Goodhead, 1994; Belli et al., 2002; Morgan, 2003a, b; Shemetun, Pilinska, 2019). Основным повреждением хромосом являются ДЦР, некорректное восстановление которых приводит к аберрантному митозу и апоптозу. В этой связи в начальных экспериментах были оценены временные параметры процесса репарации ДЦР, индуцированных γ-лучами. Мыши подвергались воздей- ствию летальной дозы облучения 9.4 Гр, клетки костного мозга вымывались через 30, 60 и 120 мин после облучения. Количество ДЦР оценивали «методом комет» или по све- чению специфических антител к гистону γH2AX (Rogakou et al., 1998, 1999; Maréchal, Zou, 2013). Результаты измерений суммированы в графике на рис. 2, а.

**Fig. 2.**
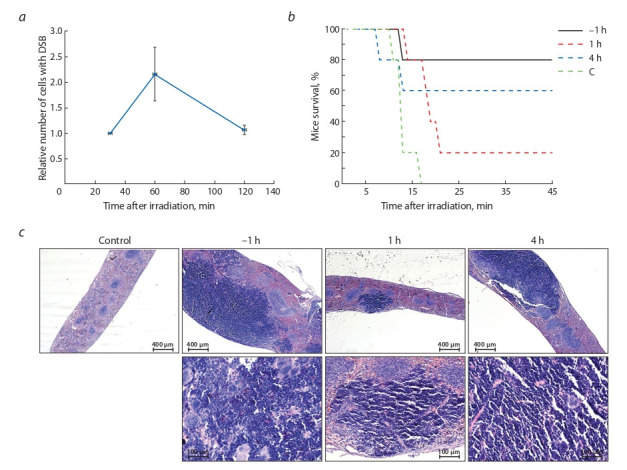
Analysis of the radioprotective effect of the RNA preparation versus the duration of double-strand break (DSB) repair. a – the duration of the DSB repair cycle in the bone marrow cells of mice after exposure to the lethal dose of γ-radiation 9.4 Gy. The graph shows the relative number of bone marrow cells in mice in which DSBs are detected; b – survival graph of experimental animals treated with the RNA preparation at critical points of the repair process, established in the analysis of the repair cycle. Mice were administered 7 mg of RNA 1 hour before irradiation (–1 h), 1 hour after irradiation (1 h), or 4 hours after irradiation (4 h), C is the control group, not treated with RNA; c – histological slides (stained with hematoxylin-eosin) of the spleen of mice on day 11 after irradiation. Control: the spleen is mostly devastated; lymphatic follicles are quite abundant but sharply reduced in size and confined to the peripheral zone of the central artery. –1 h – the major part of the organ parenchyma is occupied by a large aggregate of proliferating lymphocytes. 1 h – one large focus of lymphocytic proliferation is detected with the general decrease in the numbers of red and white pulp cells. 4 h – at least half of the section area is occupied by large foci of proliferation of lymphoid elements.

Установлено, что основной пик накопления ДЦР при- ходится на 60 мин от получения дозы радиации 9.4 Гр. К 120 мин и в более поздние сроки наблюдается практически полное восстановление целостности хроматина, тем не менее кривая не опускается до значений, полученных до облучения (данные не приводятся). Этот факт позволяет предполагать, что в указанный отрезок времени детектируются ДЦР, являющиеся интермедиатами репа- рации, идущей по механизму гомологичной рекомбинации в клетках, находившихся на момент облучения в фазе S клеточного цикла. По результатам проведенных исследо- ваний для оценки радиотерапевтического действия пре- парата РНК были выбраны временные точки 1 и 4 ч. Идея выбора состояла в том, чтобы воздействовать на клетки препаратом РНК во время наиболее интенсивного хода репаративного процесса по механизму негомологичного объединения концов в клетках, находившихся в момент облучения в фазе G1, и во время, когда возможно про- должение репарации по механизму гомологичной реком- бинации в клетках, находившихся в момент облучения в фазе S клеточного цикла (см. рис. 2, б ). Установлено, что препарат РНК обладает определенным радиотерапевтиче- ским эффектом при его введении в определенный момент времени после завершения репаративного процесса по механизму негомологичного объединения концов. Введение препарата РНК во время идущей репарации негомо- логичного объединения концов приводит к гибели мышей экспериментальной группы в стандартные временные параметры, показанные для контроля (11–14-е сутки после обработки радиацией).

Параллельно был проведен патоморфологический ана- лиз селезенок контрольных и экспериментальных мышей (см. рис. 2, в). Как показали более ранние эксперименты, основное действие препарат РНК оказывает на стволовые клетки крови, которые, пережив радиацию, мигрируют в селезенку, где формируют селезеночные колонии. Пред- полагалось, что в селезенках мышей, входящих в группы с высокой долей выживаемости, будут обнаружены центры размножения лимфоцитов, потомков выживших и мигри- ровавших в селезенку стволовых клеток крови.

Патоморфологический анализ свидетельствует о следующем. В селезенках мышей контрольной группы лимфатические фолликулы достаточно многочисленные, однако резко сокращены в размерах до периферической зоны фолликулярной (центральной) артерии. У мышей, которым ввели препарат РНК за час до облучения (груп- па «–1 ч»), большая часть паренхимы занята сплошной массой пролиферирующих лимфоцитов. У группы «1 ч», которой препарат РНК ввели через час после облучения, обнаруживается один крупный очаг пролиферации лим- фоцитов, занимающий менее 1/10 объема паренхимы. У группы «4 ч», получившей препарат РНК через 4 ч после облучения, по меньшей мере половину объема стромы занимают крупные очаги пролиферации лимфоидных элементов.

Полученный результат говорит об активной пролиферации клеточных элементов в паренхиме селезенок мышей, обработанных до облучения и через 4 ч после экс- позиции к γ-лучам.

## Сравнение эффективности радиопротекторного действия препарата суммарной РНК дрожжей и штатного радиопротектора Б-190

При сравнении эффективности радиозащитного действия радиопротектора Б-190 и препарата РНК в одном экспе- рименте (рис. 3, а) установлено, что препарат суммарной РНК обладает ярко выраженным радиопротекторным действием, не уступающим по эффективности штатному препарату Б-190. Выживаемость на 40-е сутки после облу- чения для группы мышей, получавших препарат РНК, составила 78 %, для Б-190 – 67 % животных.

**Fig. 3.**
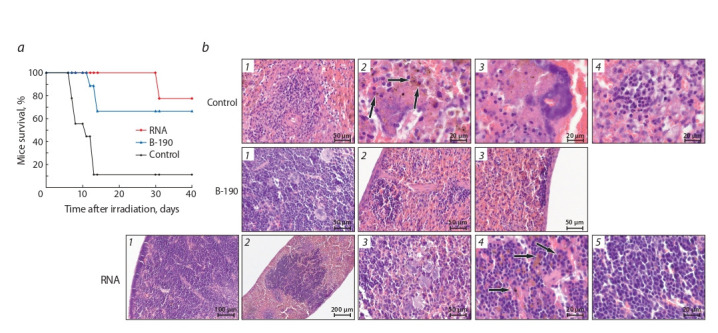
Comparison of the radioprotective effects of B-190 and the total RNA preparation. a – survival of animals after irradiation with a dose of 9.4 Gy. b – light microscopy. Control: 1 – The lymphatic follicle is reduced to the size of the periarterial zone, 2 – numerous siderophages (arrows) against the background of lysed red blood cells, 3 – aggregate of bacterial cells, 4 – hematopoietic islet in the red pulp. B-190: 1 – The spleen parenchyma is densely filled with hematopoietic blast elements, lymphoid follicles are absent, megakaryocytes are present in abundance, 2 – subcapsular location of the hematopoietic islet, 3 – accumulation of blast hematopoietic elements under the spleen capsule. RNA: 1 – Pronounced reduction of white pulp follicles, subcapsular concentration of blast hematopoietic cells in the dense layer along the contour of the left side, 2 – a large aggregate of blast elements in the central part of the parenchyma, 3 – a group of megakaryocytes, 4 – numerous siderophages among blast elements (arrows), 5 – young lymphopoietic cells.

Был проведен патоморфологический анализ селезенок и трех отделов кишечника мышей, взятых из групп суммар- ной РНК и Б-190 (см. рис. 3, б ). Ткани и органы забирались на 11-е сутки после проведенного облучения в абсолютно летальной дозе 9.4 Гр. Значимых патоморфологических изменений в эпителии кишечника экспериментальных мышей не обнаружено.

В селезенке мышей контрольной группы наблюдались только отдельные бластные клетки, лежащие небольшими островками среди сохранившихся клеток стромы. У жи- вотных обеих опытных групп, суммарной РНК и Б-190, в селезенке отмечено большое количество эритроидных клеток как в просвете сосудов, так и в паренхиме, при этом значительную их часть составляли молодые клетки кроветворной ткани, располагавшиеся в виде различного размера колоний. Таким образом, в опытных группах в селезенке наблюдалась картина экстрамедуллярного ге- мопоэза с образованием колоний кроветворных клеток, большинство из которых являлись предшественниками эритропоэза.

В отличие от мышей, профилактически получавших препарат Б-190, в группе животных, пролеченных пре- паратом суммарной РНК, отмечены выраженная про- лиферация клеток лимфоцитарного ростка и отдельные клетки-предшественники или небольшие колонии клеток других ростков гемопоэза. В селезенке мышей, получав- ших препарат суммарной РНК, присутствовали бластные клетки-предшественники миело- и лимфопоэза, многочис- ленные мегакариоциты. Предшественники лимфоцитов были преобладающим типом клеток в большей части полей наблюдения при ТЭМ исследовании. Кроме того, среди мезенхимальных клеток идентифицированы мелкие группы клеток-предшественников гранулопоэза. Одно- временно наблюдалась стимуляция фагоцитоза клетками стромы и увеличение васкуляризации органа.

Полученные результаты в большей мере предполагают, что два препарата обладают различными механизмами радиозащитного действия. В случае Б-190 защищаются клетки эритроидного ростка кроветворения. В случае пре- парата РНК и эритроидный, и лимфоидный росток кро- ветворения сохраняют свой функциональный потенциал.

## Радиопротекторное действие двух фракций дрожжевой РНК

Аналитическое исследование препарата суммарной РНК дрожжей свидетельствовало, что в препарате присутству- ют две четко разграниченные фракции, одна из которых элюируется с ГАП как одноцепочечная РНК при элюции 0.15 М PBS. Вторая фракция элюируется в условиях, характерных для двуцепочечных нуклеиновых кислот, при 0.25 М PBS. Размер элюирующихся нуклеиновых кислот находился в пределах 50–400 п. н. (рис. 4, а, б ). Проведенные эксперименты по радиопротекции обеих фракций свидетельствовали, что при равных количествах радиопротекторные свойства характерны только для фрак- ции, элюирующейся в 0.25 М PBS. При этом количество вводимого препарата, необходимое для радиопротектор- ного действия, многократно сокращалось. Если для до- стижения 80–100 % радиозащитного эффекта требуется 7–10 мг препарата РНК на мышь, то при использовании фракции, элюирующейся в 0.25 М PBS, количество пре- парата, равное 160 мкг на мышь, полностью защищает животное от абсолютно летальной дозы γ-облучения (см. рис. 4, в).

**Fig. 4.**
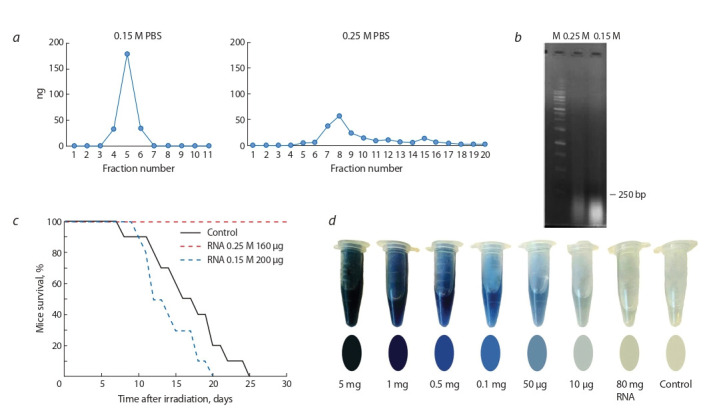
Identification of the fraction determining the radioprotective effect of total yeast RNA. a – Chromatography of total yeast RNA on hydroxyapatite. The graphs show the elution profiles of nucleic acids in 0.15 M and 0.25 M PBS; b – Electrophoretic analysis of the 0.15 M and 0.25 M nucleic acid fractions in 1% agarose gel, ethidium bromide staining, M, 1-kb molecular weight ladder; c – Radioprotective effect of the total RNA preparation fraction obtained by elution from hydroxyapatite with 0.25 M PBS. CBA mice were treated with RNA preparations 40 minutes before irradiation; d – Assessment of DNA content in the preparation of total yeast RNA by the Dische assay. The figure shows the results of the color reaction with various amounts (10–5000 μg) of DNA preparations compared to a nucleic acid preparation obtained by hydrolysis of 80 mg of total yeast RNA. The upper part of the figure shows color images of the samples after the Dische reaction. The lower part of the figure shows the colors of the samples obtained in comparison with colors of the Pantone scale. The reaction buffer was used as reference.

Одним из интригующих вопросов, касающихся фрак- ции дрожжевой РНК, обладающей радиопротекторным действием и элюирующейся в условиях, характерных для двуцепочечных структур ДНК или РНК (0.25 М PBS), был вопрос о типе нуклеиновых кислот этой фракции. В этой связи нами проведены эксперименты по характеристике молекулярного состава данной фракции суммарной РНК дрожжей. Для исследования, как и для экспериментов по радиопротекции, фракционирование нуклеиновых кислот препарата дрожжевой РНК осуществляли методом адсорб- ционной хроматографии на колонке с ГАП. Определено, что в препарате суммарной РНК присутствует ~1–3 % нуклеиновых кислот в двуцепочечной форме.

Для определения типа нуклеиновых кислот фракций были выполнены различные эксперименты с использова- нием обработки нуклеазами (ДНКаза I, SI нуклеаза) после денатурации или щелочью, или кипячением, или без тако- вой, которые не дали однозначно трактуемых результатов. В результате, чтобы установить принадлежность анали- зируемой фракции к тому или иному типу нуклеиновых кислот, был выбран метод анализа нуклеиновых кислот с использованием дифениламина и специфической цветной реакции на дезоксирибозу. Предполагалось, что если в исходной РНК присутствует ~1–3 % двуцепочечной фор- мы нуклеиновых кислот, то при выделении из большого количества исходного препарата РНК (50–100 мг) будет получена уверенная, однозначно трактуемая цветная реакция. Исходный препарат РНК в количестве 80 мг гидролизовали 24 ч слабой щелочью. После гидролиза прово- дилось осаждение полимерной формы нуклеиновых кислот. Полученные результаты свидетельствуют, что фрак-ция препарата суммарной РНК, элюирующаяся в 0.25 М фосфатном буфере, является двуцепочечной формой РНК (см. рис. 4, г), и, таким образом, можно полагать, что радиопротекторный эффект обусловлен молекулами двуцепочечной РНК.

**Анализ нуклеотидных последовательностей фрагментов двуцепочечной РНК фракции 0.25 М**

Для понимания происхождения РНК фракции 0.25 М было необходимо определить принадлежность составля- ющих двуцепочечных РНК фрагментов к генетическому локусу хромосом дрожжей. Фрагменты двуцепочечной РНК фракции, элюирующейся 0.25 М PBS, клонировали и секвенировали. Последовательности были объединены в группы гомологий (рис. 5). Анализ последовательностей секвенированных клонов свидетельствует, что в популя- ции выделяемых молекул присутствуют различные типы РНК, относящиеся к РНК рибосомального кластера или к транскриптам, кодирующим белки, ассоциированные с рибосомами. Приведенные данные предполагают, что для радиозащитного действия нуклеотидные последователь- ности фрагментов РНК не имеют значения.

**Fig. 5.**
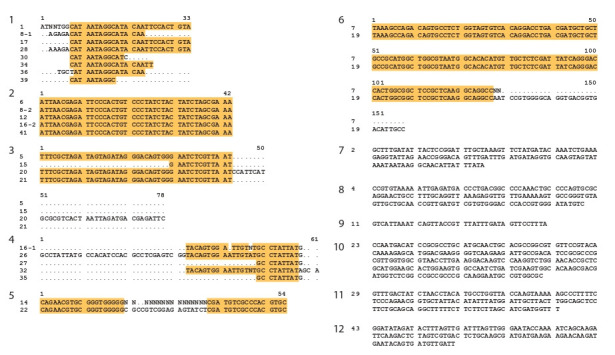
Determination of the double-stranded RNA structure. Homology groups and species affiliation of sequences: 1, 4–8, 10, 12 – homology groups or species are not defined; 2, 3 – S. cerevisiae ribosomal RNA genes RDN25-1 and RDN37-1; 9 – S. cerevisiae ribosomal RNA genes RDN18-1 and RDN37-1; 11 – S. cerevisiae TMA22 (YJR014W) gene (ribosome-associated protein).

С помощью программы https://eu.idtdna.com/calc/analyzer была проанализирована возможность секвени- рованных РНК образовывать шпилечные структуры. Для этого были выбраны наиболее протяженные последова- тельности из каждой группы гомологий. В результате обнаружено, что все проанализированные последователь- ности могут формировать шпилечные структуры. Для многих вариантов энергия образования шпилек (энергия Гиббса) имеет высокое значение, что предполагает пре- имущественное формирование таких структур (данные не приводятся).

## Обсуждение

В нашей ранней работе (Likhacheva et al., 2007) было пока- зано, что фрагментированная ДНК (препарат «Панаген», ЛСР № 004429/08 от 09.06.2008, ДНК мыши), введенная в организм смертельно облученных мышей, обладает выраженным радиопротекторным действием – при ЛД100/30 выживаемость животных составляет 70–90 %. При этом радиопротекторный эффект фрагментов двуцепочечной ДНК коррелирует с развитием селезеночных колоний. Кроме того, при внутривенном введении экстраклеточная двуцепочечная ДНК доставляется в клетки костного мозга, в том числе в CD34+ стволовые гемопоэтические клетки мыши, где может депонироваться и обнаруживается в течение 14 дней после введения (Dolgova et al., 2013a, b). Именно эти два факта легли в основу предположения, что фрагменты двуцепочечной ДНК спасают стволовые гемо- поэтические клетки, которые мигрируют на периферию, стабилизируются в селезенке и дают начало новой кро- ветворной и иммунной системам мышиного организма, разрушенным высокодозовой γ-радиацией.

Поскольку было показано, что двуцепочечная форма нуклеиновых кислот (ДНК) отвечает за радиопротектор- ный эффект, детектируемый в проведенных эксперимен- тах, нами сделано предположение, что за радиопротекторный эффект препарата суммарной РНК S. сerevisiae отвечает присутствующая в нем двуцепочечная фракция нук- леиновых кислот.

Хроматографией на гидроксиапатите была выделена фракция препарата РНК, элюирующаяся с колонки как двуцепочечная форма нуклеиновых кислот. Биологиче- ские тесты на радиопротекторные свойства этой фракции однозначно свидетельствовали, что за радиопротекторный эффект препаратов РНК дрожжей отвечает двуцепочечная форма нуклеиновых кислот, составляющая ~1–3 % от сум- марной РНК препарата, находящегося в работе. При этом эффективная доза ЛД100/30 для суммарного препарата со- ставляла 7–10 мг/мышь, в то время как для двуцепочечной формы – 160 мкг/мышь, что в ~60 раз меньше. В многократных экспериментах показано, что введение препарата двуцепочечной нуклеиновой кислоты за 60–30 мин до облучения полностью купирует радиационное действие γ-потока. Выживает 80–100 % экспериментальных мышей.

С использованием метода дифференцированного гидролиза щелочью и кислотой и специфического качественного окрашивания на присутствие ДНК было установлено, что двуцепочечная форма, выделяемая в составе препаратов РНК и обладающая радиопротекторными свойствами, представляет собой двуцепочечную РНК. Фрагменты двуцепочечной РНК, переведенные в форму кДНК, были клонированы и секвенированы. Определено, что смесь фрагментов двуцепочечной РНК гетерогенна по первич- ной структуре и, по-видимому, для осуществления радио- протекторного действия не требуется специфической по-следовательности.

При анализе радиозащитного действия двуцепочечной РНК установлено, что так же, как и в случае с препаратами двуцепочечной ДНК, в селезенках экспериментальных животных формируются селезеночные колонии. Колонии состоят из пролиферирующих клеточных элементов, кото- рые, как предполагается, представляют собой потомков, спасенных стволовых гемопоэтических предшествен- ников, дающих начало новой кроветворной и иммунной системе, которые были разрушены облучением.

Введение препарата в точку максимально активной репарации ДЦР, идущей по механизму негомологического объединения концов, через 1 ч после получения полной летальной дозы 9.4 Гр (см. рис. 2, б ) не защищает мышей от гибели от облучения. При этом инъекции препарата через 4 ч после облучения, т. е. тогда, когда активная фаза процесса репарации негомологичного объединения кон- цов завершена, эффективно (до 60 %) спасают мышей от гибели. У таких животных полностью восстанавливаются кроветворные ростки костного мозга и детектируется вы- раженное колониеобразование в селезенках.

Предполагается, что фрагменты двуцепочечной РНК, доставленные в клетку в момент идущего репаративного процесса, интерферируют процесс репарации негомоло- гичного объединения концов, причем эта интерферен- ция может быть обусловлена различными механизмами (конкурентное связывание репаративных комплексов, индукция конфликтного репаративного процесса иной природы, блокада квазиматрицей субстратных двуцепо- чечных концов).

Облучение с указанной дозой 9.4 Гр и мощностью 0.74–1.4 Гр/мин является острым облучением, для кото- рого появление и накопление ДЦР, оцененное методом фокусов к гистону γH2X, происходит к 40–60-й минуте после окончания облучения (Peitzsch et al., 2013; Озеров, Осипов, 2015). Такой результат близок к данным, полу- ченным в настоящем исследовании.

Известно, что при остром облучении помимо простых ДЦР формируются «сложные», образующиеся в результа- те индукции других повреждений хроматина и активации иных репаративных процессов (Озеров, Осипов, 2015). Авторы цитируемой работы сообщают, что до 20 % ДЦР при γ-облучении относятся к «сложным повреждениям» и репарируются значительно позже, чем ДЦР, индуци- рованные непосредственным разрывом хроматина. Воз- можно, обнаруженный терапевтический эффект связан с репарацией хроматина в стволовых клетках костного мозга по типу гомологичной рекомбинации с участием внешней РНК матрицы. Этот тип репарации активируется значительно позже по сравнению с аварийным не- гомологичным объединением концов. Тот факт, что на графиках, полученных при оценке числа ДЦР, показатели в последней анализируемой точке (2 ч) ни в одном из приведенных экспериментов не опускались до значения исходной нулевой отметки, согласуется с высказанным выше предположением.

В литературе известны варианты репаративных процес- сов с использованием РНК и ДНК матрицы. Для РНК опи- саны модели, в которых основным лейтмотивом является построение кДНК копии и вовлечение двуцепочечной формы этой нуклеиновой кислоты в репаративный про- цесс (Storici et al., 2007; Meers et al., 2016). Для двуцепо- чечной ДНК также известны различные модели репарации с привлечением внешней двуцепочечной матрицы (Leung et al., 1997; Bärtsch et al., 2000; Li et al., 2001; Symington, 2005). Характерным для участия таких нуклеиновых кис- лот в идущем репаративном процессе является внедрение процессированного 3′OH конца разорванного хроматина между цепей внешней матрицы и формирование интерме- диата репарации. Далее могут осуществляться различные описанные варианты достраивания цепей и восстановле- ния целостности хроматина. Можно предположить, что репарация в присутствии экстраклеточных двуцепочечных РНК идет именно по такому общему молекулярному сце- нарию. О важной роли двуцепочечной формы нуклеино- вых кислот при осуществлении репаративного процесса свидетельствуют данные, полученные в работе (Storici et al., 2007), где показано, что дуплекс РНК/ДНК повышает эффективность репарации по сравнению с одноцепочеч- ной РНК на два-три порядка.

Полное отсутствие радиозащитного действия у препарата одноцепочечной РНК в дозах, сопоставимых с радиопротекторными дозами двуцепочечной РНК, предпо- лагает, что радиопротекторное действие двуцепочечной РНК связано с появившейся в клеточном пространстве стволовых гемопоэтических клеток внехромосомной двуцепочечной матрицы. Внедрение между цепями такой дву- цепочечной РНК матрицы процессированного филамента ДНК ДЦР может быть главным событием, определяющим дальнейшие фазы репарации фатального повреждения, индуцированного γ-радиацией.

## Заключение

Таким образом, данные молекулярно-биологических ис- следований, экспериментов с использованием клеточных технологий и биологические тесты свидетельствуют, что субстанцией, определяющей радиопротекторное действие фракции «0.25 М» РНК дрожжей S. сerevisiae, является двуцепочечная форма молекул РНК.

## Conflict of interest

The authors declare no conflict of interest.
